# Inter‐professional practice in the prevention and management of child and adolescent self‐harm: foster carers’ and residential carers’ negotiation of expertise and professional identity

**DOI:** 10.1111/1467-9566.13071

**Published:** 2020-04-14

**Authors:** Stephen Jennings, Rhiannon Evans

**Affiliations:** ^1^ Centre for the Development and Evaluation of Complex Interventions for Public Health Improvement Cardiff University Cardiff UK

**Keywords:** Qualitative, Self‐harm, Interprofessional Practice, Interprofessional Education

## Abstract

Inter‐professional collaboration remains a significant concern within healthcare and social care. However, there has been scant attention paid to practices at the interface of clinicians and carers, namely foster carers and residential carers. The present study considers child and adolescent self‐harm management and prevention practices as a site of empirical interest due to reports that multi‐agency teams are not effectively operating. Drawing upon a grounded theory approach, data were generated via semi‐structured interviews and focus groups with residential carers (*n* = 15) and foster carers (*n* = 15) in Wales. Themes were developed through axial coding. The results present two central themes to explain the nature and perceived causes of inter‐professional discord. First, there are clear contestations in expertise, with carers challenging clinicians’ propositional knowledge in favour of their own experiential expertise. However, participants simultaneously endorse medical dominance, which contributes to their sense of disempowerment and marginalisation. Second, is the preclusion of carers’ professional identity, primarily due to inadequate professionalisation procedures. Meanwhile, the privileging of their parenting role is perceived to support the perpetuation of courtesy stigma. Carers are then compelled to undertake the effortful labour of legitimisation. Together these thematic insights provide direction on mechanisms to improve inter‐professional interactions, notably around training and accreditation.

The intricate dynamics of inter‐professional communication and collaboration remains of central interest within healthcare and social care. Both *in situ* and reflective accounts of practices document seemingly entrenched challenges (Best and Williams 2019), including differences in professional vocabulary, inadequate resources and lack of commonality in outcome goals (Glaser and Suter 2016, Karam *et al*. 2018, Mitchell *et al*. 2011). These factors often intersect with and are informed by a complex nexus of social structures, such as class and gender, which can support the development of distinct and even incommensurable professional cultures (Hall 2005). Inter‐professional education (IPE) programmes have sought to introduce and even standardise pedagogic approaches to enable collaborative working and support the exchange of skills and knowledge (Chen *et al*. 2019, Gilbert *et al*. 2008, Steven *et al*. 2017). In potential tension with these integration efforts, however, are processes of ‘boundary work’, where individuals and groups actively engage in distinction practices in order to create demarcation and distance from other professionals (Allen 1997, 2000, Burri 2008, Gieryn 1983). Motivations here are multi‐faceted but can often centre on the need to elevate status through verifying a unique expertise and contribution.

While this extant research on inter‐professional practice is both conceptually and empirically instructive, to date it has remained somewhat limited in its focus. Considerations of interactions across agencies have tended to centre on the intersection of health and social care, where social care is often reduced to affiliated health professionals (O'Carroll *et al*. 2016). As such, there has been more limited attention to the interface with a diverse range of social care actors, notably those individuals responsible for providing non‐permanent out‐of‐home care for children and adolescents, such as foster and residential carers. Within the UK context, foster placements entail a child living in the carer's home and can be provided as an emergency, short‐term or long‐term option. Meanwhile, residential care can encompass both secure and non‐secured children's homes, with the former having a particular focus on providing care for those at risk of harming themselves and others. Where we do see such actors feature in studies around inter‐professional collaboration, they have often been limited to discrete cases of child protection procedures (Hood *et al*. 2014).

Attending to inter‐professional relationships with this group is necessary, as there exists a particular and historical set of power dynamics and circumstances that may serve to render interactions across professional disciplines problematic. Most fundamentally has been tensions around the notion of the ‘professional’ carer, with debate over whether this group can even be defined as ‘professionals’, and thus in possession of a professional expertise. This contestation may be partly attributed to care being conceived as a largely voluntary activity (De Wilde *et al*. 2019, Vanderfaeille *et al*. 2016, Wilson and Evetts 2006). In fact, the inadequacy of remuneration has potentially reinforced the perception of caring as being altruistically motivated (Colton *et al*. 2008). This belief has arguably been compounded by the fact that despite being statutorily delineated, the caring role necessitates the blurring of the personal and professional; professional status is contingent on the ability to carry out the intimate, everyday task of nurturing a child (Schofield *et al*. 2013, Thompson and McArthur 2009). As such, the fulfilment of conflicting demands can create an impossible space for carers who must simultaneously fulfil the duties of professional and parent, while potentially being precluded from fully embodying either role.

Recent efforts have sought to instigate professionalisation processes in foster care, with a particular focus on the re‐distribution of power from social workers. However, these attempts have been considered to give rise to a range of unintended harms. Notably, Wilson and Evetts (2006) observe that professionalisation of carers has simply magnified scrutiny by other groups through the exacerbation of regulation and accountability. Rather than seeking to privilege the status of carers then, the process of professionalisation actually serves to corral it within the wider discourses of social care, thus embodying the Foucauldian notions of surveillance and control (Foucault 1977). Given that foster carers already report feeling disempowered, mistrusted and undervalued (Blythe *et al*. 2011, Gilbertson and Barber James 2003, Rosenwald and Bronstein 2008), increased opportunities to commodify and critique their contribution may only then serve to aggravate such sentiments, creating resentment and reifying tensions between agencies. Within this context then, it is imperative to understand any particularities of working at the inter‐professional boundary within this discretely constructed group.

Self‐harm prevention and management offers an empirically interesting phenomenon through which to explore relationships between healthcare and these specific social care professionals. NICE guidance has recognised the integral role of effective multi‐agency team collaboration in promoting the mental health and wellbeing of care‐experienced children and adolescents (National Institute for Health and Care Excellence 2010). Efforts to standardise and improve such an approach have become imperative where structures to support inter‐professional working are not considered to be working effectively (House of Commons Education Committee 2016, York and Jones 2017). Accounts of such failings in this area have historically centred on the lack of time or inadequacy of access across agencies (Stanley 2007).

More recently, however, there has been suggestion that challenges may be partly due to discordant terminology and incongruence in understanding across professions (Cooper *et al*. 2016). Self‐harm is particularly vulnerable to these issues, as it arguably possesses a degree of interpretive flexibility due to ambiguity over motivations to engage in such practices. Evans’ (2018) recent exploration of social care professionals’ understandings of the causes of self‐harm reveals a set of interpretations grounded in the complex relational dynamics associated with a young person being in care. These socioculturally informed interpretations may be juxtaposed with clinicians’ perceived reliance on a bio‐medical model of causation, which has been explored elsewhere (Chandler 2014, 2019, Chandler *et al*. 2011). These tensions can also be located within wider considerations of clinicians reported attitudes towards individuals presenting with self‐harming practices, which have tended to be negative and position ‘patients’ as making an illegitimate claim on professional expertise (Saunders *et al*. 2012, Taylor *et al*. 2009). Therefore, it is important to attend to the possibility that social care professionals may engage in distinction practices in the effort to demarcate their differences from those who have been unfavourably characterised for their professional practice.

Drawing upon interview data generated with foster and residential carers in Wales, the present paper explores inter‐professional relationships between social care and healthcare professionals, utilising self‐harm prevention and management as a site for study. Identifying fractures in inter‐professional collaboration, it explores two central driving factors. First, is the contested notion of expertise; carers simultaneously endorse the technical skill and knowledge of clinicians as signifying expert status while disputing the legitimacy and validity of this expertise as experientially informed. In undertaking distinction practices, carers exacerbate the relegation of their own professional knowledge, while expressing frustration and disappointment at this position. Second, is carers’ perceived ascription of a diminished and even disenfranchised professional identity by clinicians, which is sustained through the process of courtesy stigma and the inadequacy of professionalisation processes. Carers are then compelled to undertake the effortful labour of legitimisation.

## Research design

The study design adheres to a grounded theory approach (Glaser and Strauss 1967, Strauss and Corbin 1998), with the aim of generating theoretical insights into how self‐harm is understood and managed across healthcare and social care settings. The findings pertaining to social care‐professionals' construction of self‐harming practices are presented elsewhere (Evans 2018). The present data explore the wider inter‐professional context of working practices.

### Sample and recruitment

Data were generated with 30 carers across Wales who have a statutory responsibility for children and adolescents aged 18 years or younger. Participants were foster carers (*n* = 15) and residential carers (*n* = 15). These were sampled due to being the most prevalent care type within the UK context. Of the 4715 children and adolescents residing in out‐of‐home local authority care in Wales during the study period of 2015–2016, the majority were in foster care (*n* = 4365) or local authority/private residential care (*n* = 250) (StatsWales 2016). During the same period, there were 63,660 children and adolescents in out‐of‐home care in England (Department for Education 2016), 1933 in Northern Ireland (Northern Ireland Department of Health 2017) and 11,447 in Scotland (Scottish Government 2017), with fostering being the primary placement type across all nations. Foster care and residential care also provide an interesting analytical contrast due to historic perceptions of residential care being the ‘last resort’ for acute cases, although this assertion has been increasingly critiqued (Elliott *et al*. 2017).

Twenty‐three of the professionals were female and seven were male. Ten participants had up to 5 years of experience of caring for children and adolescents, 12 had 6–10 years of experience, and 8 had more than 16 years of experience. Nineteen provided generic foster care or residential care placements, while 11 offered specialist placements for adolescents exposed to particular types of maltreatment or having additional physical, behavioural, or emotional needs. Twenty‐nine participants had direct experience of self‐harm among children and adolescents, with one individual discussing preparedness to work collaboratively.

Participants were purposively recruited through a private foster care association, a national foster carer network, and a private residential care association comprising of a large number of group homes. Each of these associations disseminated information about the study to members via email or a meeting. The information invited members to participate in a focus group on a pre‐specified date or to contact the lead researcher [RE] to arrange an interview. The recruitment of participants was conducted until theoretical saturation was approached (Charmaz 2006, Saunders *et al*. 2018), whereby we felt we had achieved a rich and nuanced understanding of activities and experiences related to carers’ inter‐professional practices. Specifically, we had achieved representativeness and consistency in key emerging concepts that supported explanation of practices, such as ‘contested expertise’ or ‘labour of legitimisation’.

### Methods

Focus groups and semi‐structured interviews were undertaken with participants. Nine participants took part in interviews. Three were undertaken in person and six via telephone. Focus groups were conducted with 21 carers. The intention was to conduct focus groups only, as the research was interested in exploring both intra‐professional interactions *in situ* and how social care professionals understood and negotiated inter‐professional relationships. However, due to the sensitive nature of the subject matter we acknowledged that some participants may find it difficult or distressing to discuss self‐harm in an open forum, and thus interviews were offered as an alternative. While maximising opportunity for study participation, the mixing of qualitative methods is not unproblematic, particularly in regard to the different research questions they can answer and the different types of data they may generate (Barbour 1998, Padgett 2016).

The topic guide explored: carers’ lived experiences of self‐harm and suicide among the children and adolescents they care for; existing prevention and management strategies, including perceptions of expertise and inter‐professional working; and future prevention and management needs. Data collection was led by the primary researcher [RE]. Data generation and analysis were undertaken concurrently, with the topic guide being refined and developed as themes emerged. Data were transcribed verbatim by a professional transcription service with specialist expertise in sensitive topics and reviewed for accuracy. Data were collected between November 2015 and May 2016.

### Ethical procedures

Participants were provided with information about the study in advance of data collection, documenting confidentiality, anonymity and the process of informed consent. The researcher and participant discussed the information prior to providing consent, to ensure any questions or uncertainties were addressed. Written consent was provided by individuals participating in in‐person interviews with verbal consent obtained for telephone interviews. Data were recorded with an encrypted audio‐recorder, before being transferred to the secure University network. All participants were ascribed a pseudonym. Data are retained and archived in accordance with Cardiff University's retention schedule.

Although the extant evidence suggests that discussing suicide tends not to confer significant harm or distress (Blades *et al*. 2018), participants were provided with a list of resources relating to self‐harm and suicide that they could follow‐up with in the event they required additional support. At the end of each interview participants were offered a follow‐up, debrief call. Support for the primary researcher was provided through routine supervision with an academic colleague. Ethical approval for the study was provided by Cardiff University School of Social Sciences Ethics Committee.

### Analysis

In accordance with the grounded theory approach (Glaser and Strauss 1967, Strauss and Corbin 1998), analysis commenced with an ‘open’ reading of the data to code the text, with a coding framework being developed and confirmed with a subset of data. The framework was developed and refined as coding progressed. Coding was undertaken by SJ and verified by RE, with discrepancies over interpretation being resolved through discussion. At this point in analysis, focus group and interview data were treated independently in order to explore differences in the corpus of data according to the methods employed to generate them. Memos were recorded through this and subsequent analytical phases. The proprietary qualitative analysis software package NVivo 10 (QSR International, Melbourne, Australia) on Windows was utilised.

Axial coding was then conducted in accordance with the four constituent elements. First, codes were categorised according to the phenomenon under consideration (e.g. inter‐professional relationships). Second, codes were examined for those explaining the conditions that give rise to the phenomenon (e.g. expertise). Third, categories of codes were developed to explain the practices and experiences related to the management of the phenomenon (e.g. inter‐professional communication). Fourth, categories explored the consequences of these actions (e.g. experiences of marginalisation within inter‐professional interactions).

The process of analysis involved the continual revisiting of the data in order to re‐contextualise and further develop themes from the four categories. Some themes were collapsed or expanded through comparison. Two super‐ordinate themes emerged that appropriately represented the data around inter‐professional practices. Within these themes theoretical constructs were developed. Importantly, narratives here were not treated as objective accounts, but rather social constructions that formed part of the narrators’ identity work (Gubirum and Holstein 2002). Of note, attention was paid to how participants often used the rhetorical device of indirect complaining about third parties (Edwards 2005) in the process of constructing notions of professional status. Particular attention was paid to differences in themes according to the method employed. While there was some variation in the extent of discussion around intra‐professional relationships, significant differences in the use of rhetorical devices or identity work in regard to inter‐professional practices across methods were not observed. To minimise bias emergent and final themes were interrogated and confirmed by both authors.

## Results

The following results comprise two sections, with each exploring relationships at the interface of social care and healthcare professionals. While many accounts refer to specific interactions around child and adolescent self‐harm management, participants often drew upon wider experiences of inter‐professional practice in order to illustrate their perspectives and arguments. The first section unpacks carers’ understanding of contested expertise, notably the perceived duality and potential incongruence between their tacit, experiential knowledge and the formal, propositional knowledge of clinicians. The second section considers the legitimacy of carers’ professional identity, and how they feel disenfranchised through the process of courtesy stigma and the inadequacy of professionalisation processes. Together these empirical accounts help to develop the theoretical insights into how tensions emerge and endure between professional groups.

### Contestations in expertise: the duality of propositional and experiential knowledge

Accounts of inter‐professional working repeatedly centred on the duality of expertise and knowledge considered to be in operation. Interactions with clinicians often brought into sharp relief the divergences in understanding of self‐harming practices, with participants juxtaposing their sociocultural understandings with the bio‐medical discourse deployed by medical professionals (see Evans 2018). This professional distance was compounded by the fact that clinicians were seen to be in possession of a formalised, technical expertise that was based on propositional knowledge. Participants often described clinicians’ practice as drawing on conceptual schemas, as embodied in diagnostic frameworks. In some instances, their practice was seen as so reliant on abstracted knowledge that they did not even need to have a direct encounter with the adolescent in question in order to exercise their expertise. For example, one residential carer communicated exasperation as to the practice of clinicians offering a diagnosis in the absence of the child:

I've known CAMHS workers to come out, doctors from CAMHS to come here and the young person wasn't here, and they spoke to me and a member of staff and give a diagnosis on what was wrong with that young person. They haven't even spoken to them. Well how can you do that? (IDRC04: Residential Carer)

Carers even went so far as to suggest that ‘real’ knowledge of a young person necessitated going beyond a direct observation of them and required an understanding of how the individual in question interacted with the world around them. Professionals’ efforts to know and assess adolescents led to young people drawing upon the technical information and concepts to navigate and negotiate the system. Indeed, some participants shared examples where they felt young people had drawn upon rudimentary medical knowledge and presented themselves to clinicians in a particular manner (i.e. having poor mental health) in the effort to gain some advantage in a system that routinely disempowers them:

They might have learned it, they might have worked it out for themselves. We're seeing in this child, if you talk to her about self‐harm. She reads the internet. She knows what she's supposed to say. So, she will say all the stuff about it being a release and this and that because she's read that's what self‐harm is about and you find that [pause], um, professionals can be taken in by this because she's ticking the boxes. (IDRC04: Residential Carer)

Study participants, including the residential carer above, sought to distance themselves from the expert identity that they associated with clinicians, and what they considered to be a limited, reductionist and often illegitimate form of knowledge. They positioned themselves as having an understanding of the complexity of adolescents’ lives, and also how these individuals could use their agency within relationships with professionals to gain control over how knowledge about them was constructed and used:

… There is [pause] this mantra that you always have to believe children. And if, they don't actually realise that these children have had to look after themselves to keep themselves alive for say 15 years. These children are streetwise. These children are manipulative and the children do not always tell the truth. (IDRC03: Residential Carer)

For participants, there was a clear focus on privileging this deeper, nuanced understanding of adolescents. This tacit knowledge was routinely characterised as experiential, and could be acquired though everyday intimacy with a young person:

But it's not authentic [how adolescents present to clinicians]. And I think it's because we pay so much attention to the child that you, you know, you can see that. (IDRC04: Residential Carer)

Yeah but I think as well you can have as much training as you want. You know you can sit and be lectured but actually you learn from experience. (IDRC14: Residential Carer)

Participants claimed to routinely engage in distinction practices in order to demarcate their differences from clinicians, which extended to include the explicit questioning of their knowledge about the young person and their understanding of the wider context of the young person's life. Yet, despite apparent factions, and carers' clear efforts to distinguish different forms of expertise, at times the degree of complexity of the problem in question (i.e. self‐harm) made their reliance on clinicians’ expertise somewhat necessary. In fact, as explicated in studies of inter‐professional relationships around child protection (Hood *et al*. 2014), at a point complex cases can require specialist support and intervention where they risk feeling stuck or intractable.

This sense of necessity gave rise to more nuanced forms of inter‐professional tension than a belief in the incongruence of expert knowledge. In feeling compelled to seek and even defer to clinicians’ decisions in complex cases of self‐harm management, participants’ narratives implicitly constructed medical professionals’ expertise as the normative referent for the ‘expert’. This set of structures seemed to diminish carers’ own sense of professional status, causing feelings of disenfranchisement and doubt about their own capabilities. As one residential carer suggested, ‘*like I said I'm no expert* [on self‐harm] *so I just have to go on what I was being advised to do’*.

Instances where clinicians were invited into carers’ spaces in a specialist capacity were often experienced negatively, with medical professionals often being characterised as dominant or overbearing:

Because I wasn't able to put a name on what I think it [self‐harm] could have been or you know, suggest what it may have been and push a little bit further, I felt quite overpowered by these big psychologists and doctors, that it was kind of a bit, like no, it's nothing really. (IDRC10: Residential Carer)

One residential carer commented on how they were made feel inadequate and insignificant:

Like I say the last time I dealt with CAMHS I just felt that they were talking down their nose at you. Looking down their nose at you. Who are you? What do you know? (IDRC04: Residential Carer)

Through the seeming necessity of clinicians’ situational dominance in such instances then, there is apparently scant opportunity to move beyond the duality of expertise and towards spaces where a plurality of knowledge is privileged. The endurance of this binary places carers in a rather passive and submissive position, and this disenfranchisement becomes a lens through which they experience clinicians’ practices.

### Preclusion of professional identity: inadequate professionalisation processes and the labour of legitimacy

Carers’ narratives explored the constant and complex negotiation of inter‐professional relationships in order to secure a professional identity on a parity with clinicians. Throughout accounts, participants cited perceived instances of clinicians’ efforts to demarcate their elevated status in relation to them. These tended to focus on carers’ systematic marginalisation from information sharing and decision‐making:

The other thing is the way the statutory agencies don't involve, I mean we are the amateurs, really aren't we? That's what they see. They don't actually kind of seem to realise the level of expertise. So, where they have the multi‐agency meetings, they will have multi‐agency meetings about our children but not invite us because we are not a statutory agency. And often we're providing, we will provide a report and they won't read it because we're not a statutory agency. (IDRC03: Residential Carer)

Explanations of clinicians’ efforts to prevent carers’ assumption of a professional identity was largely ascribed to the lack of a professional qualification for foster and residential carers, meaning they were unable to signify possession of the formalised, propositional knowledge possessed by other professional groups:

I think there's a need for a lot of foster carers to be recognised as maybe, erm, more experienced than they think… . As soon as you have the foster carer hat on, it seems as if you're not treated as professionals, so maybe a professional qualification would help there. (IDFC03: Foster Carer)

There was further consideration of the complex professional‐parent nexus, and carers struggled to frequent the space of the corporate parent. Throughout narratives were accounts of being reduced to a *‘childminder’* or *‘babysitter’,* with the parenting aspect of their role being foregrounded:

Society as a whole need to wake up and even the caring community needs to wake up to the fact that carers are now of a very high standard. When we first had the young fella, we got we took him to the local primary school and we felt we were [pause] almost dismissed as sort of sub human by the teaching staff and not seen as professionals, we were seen as childminders. (IDFC01: Foster Carer)

This construction of carers has particular relevance in regard to the area of self‐harming, as it seemingly contributed to the phenomena of courtesy stigma. Participants believed themselves to be negatively labelled by medical professionals due to their association with the presenting care‐experienced young person. They felt they were treated in a ‘*derogatory*’ or disparaging manner because they were responsible and had somehow contributed to the self‐harm due to their close relationship with the child:

We were taking a couple of boys in the hospital off to the doctors. I think the staff are looked at in a very derogatory way because it would feel as if, oh, I was thinking of Peter being taken to the hospital where the nurses would all look at us as if we've got two heads. As if you've caused this boy to [self‐harm]. (IDRC06: Residential Carer)

Such experiences need to be located within the wider discourses that participants feel imbue healthcare settings. In particular, carers maintained care‐experienced children and adolescents are constructed as problematic. This was linked to them being rendered highly visible within hospitals due to frequently having complicated health needs that are grounded in complex causes. Carers felt that healthcare and affiliated professionals quickly grew tired and frustrated by the claims such complexity placed on their time, resource and expertise, which could lead to inadequate provision:

And you can see the CAMHS workers coming, ‘Oh they're from [Residential Care Centre], they're kids in care’ … You can tell by their faces. And then they wonder the young people won't speak to them, you know. I think it's, I wouldn't call it ignorance but I think there is some of that as well. Yeah, you know, these kids have got issues and that's why they're with us. So, the chances are you're gonna see them a bit more than your normal Joe Bloggs off the streets. Maybe in hospital, you know. (IDRC04: Residential Carer)

Accounts of punitive reactions to carers accompanying adolescents who had self‐harmed to hospitals were also documented:

And we were left in the car park for 2 and a half hours while we were restraining her ‘cause she was trying to run away. We then got taken in after so many hours […] and I think you find that if you're in with a young person that's self‐harmed you don't get anything … as in you know don't worry ask me if I want a drink, you know with the sweat pouring off you … They're not very nice with them at all […] But a drink wouldn't hurt, you know to give somebody a drink. And then when we got put onto the ward we were put like into a little room, and it's just like we were an effort for everybody. It was like we were too much hard work. (IDRC05: Residential Carer)

In the absence of professional qualification and with uncertainty of their role contributing to the problem, carers felt compelled to engage in a range of practices in order to demonstrate their unique value and contribution. For many, this included sharing social care based information and knowledge with clinicians at the multi‐agency meetings they were invited to:

As they've sat in on meetings that we have been in with other care professionals on this young man, they've sort of changed and they have realised that actually [laughs] we are fellow professionals, not childminders and we have even been able to pass on leaflets and pamphlets and things to help them with the care of the young person we've got, which is good to be able to help them. We now have an excellent rapport with them. (IDFC01: Foster Carer)

Inscribed in such narratives is the apparent and problematic criteria through which carers contributions are assessed. In essence, as the above quote illustrates, carers felt their value is assured only when they have been deemed ‘useful’ or ‘helpful’ in assisting clinicians in the application of their own formal, technical knowledge. They rarely seemed afforded the opportunity to provide meaningful or extensive input on specific children but were invited to offer generic support. This situation seemed inherently unsatisfactory for participants, as any legitimacy as a professional is temporary and contingent on the specific inter‐professional interaction and carers’ capacity to meet the particular needs of the clinician. This creates somewhat of a disempowering nexus of relations for carers, having to carry the burden of proving their legitimacy and value of their contributions within each new multi‐agency interaction. We can term this burden as a labour of legitimisation.

To avoid having to continually undertake this labour, some participants sought to eschew more formalised inter‐professional interactions where possible, seeking to operate more creatively within statutory procedures. In such cases, they invested considerable time in building selective and more informal networks centred on familiarity with specific individuals who they felt they could develop a personal connection with. These relationships became so enduring that it meant carers did not have to repeatedly engage in the labour of legitimisation that would be undertaken within new collaborations, as it had already been performed. For example, one residential carer touched upon their productive working relationships with the local CAMHS teams, and as the discussion progressed it emerged that inter‐professional working was very much built on communicating with a particular nurse that they had come to know well over a number of years:

When we've had a young person from [Local Authority] who has gone to [Local Authority] CAMHS the service has been just unreal. I cannot fault them. They've been superb with us, with the young person, with the whole thing. I cannot fault them. And we worked really well with CAMHS and they listen to us and we listen to them. And I think we've built an extremely good relationship with them to the point that they think [CAMHS Nurse] is the best thing since sliced bread. (IDRC13: Residential Carer)

While such an approach provides a remedy to tensions at the inter‐professional boundary and the need to perpetually undertake the labour of legitimisation, it arguably prevents the development of new and innovative collaborations to address complex healthcare or social care problems.

## Discussion

The present study has explored the complex experiences of foster carers and residential carers working at the inter‐professional interface with clinicians. Self‐harm management and prevention served as an interesting site for empirical consideration, primarily due to current policy frameworks and guidance indicating that existing structures are not working effectively (House of Commons Education Committee 2016, York and Jones 2017). While there is a wealth of extant research explaining inter‐professional tensions, these have predominantly privileged healthcare and affiliated professionals’ interactions (O'Carroll *et al*. 2016), although there is some consideration of practices in relation to child protection procedures (Hood *et al*. 2014).

Relationships were characterised by discord and dissent, with two central processes identified as offering part explanation for this occurrence. These primarily centre on contestations and conflicts in expertise and professional status, leading to carers’ distinction practices in order to create distance or establish legitimacy (Allen 1997, 2000, Burri 2008). There was seeming incongruence between the propositional knowledge acquired by clinicians through stringent accreditation (Reeves *et al*. 2010) and the experiential expertise developed through carers’ familiarity and everyday intimacy with the individuals they care for.

One of the primary discriminating factors between these types of expertise was the understanding of children and adolescents as agentic beings. There was a sense that clinicians treated their patients as homogenous, where they could even be diagnosed without actually meeting them. In contrast, carers saw their heterogeneity as linked to the exercise of agency, to the point where they could actively assimilate and even manipulate clinicians’ knowledge in order to navigate the care system to their distinct advantage. It is this awareness of a double hermeneutic (Giddens 1984), where technical constructs can enter constitutively in the world they describe and be used by that very world, which carers felt gave them a particular warrant in decision‐making.

While exploration of the tensions in different forms of expertise is not novel, one of the oversights in previous research has been full consideration of the impact on the individual, particularly how such tensions can be reflexively assimilated into the sense of self. In being compelled to seek specialist clinician support in complex cases of self‐harm, carers often served to devalue their own expertise when reflecting on their potential contributions. In fact, in acknowledging the necessary situational dominance of clinicians in some instances, carers occasionally positioned themselves outside of any expert identity. This seemed to contribute to inter‐professional discord, as their disempowered position seemed to become a lens through which clinicians were negatively appraised as overpowering.

Carers’ beliefs that they were precluded from assuming a professional identity was a further issue elucidated. Resonating with wider documented concerns about the lack of professionalisation of foster carers (Blythe *et al*. 2011, Gilbertson and Barber James 2003, Rosenwald and Bronstein 2008, Wilson and Evetts 2006, Wilson *et al*. 2000), the study highlights how carers can feel systematically marginalised due to their lack of professional accreditation. Rather, there was a sense of being reduced to mere ‘*babysitters*’, where the parenting aspect of their role is foregrounded. Most importantly, and rarely brought to the fore within the extant literature, is the effort entailed in navigating identity from such a disenfranchised position. Indeed, research has tended to describe the process and value of obtaining legitimacy within a particular nexus of power relations (Ehrlich *et al*. 2019, Friedson 2007), often failing to explicitly recognise the effortful labour involved in this legitimisation. Elicitation of this labour, which can entail proving value against a criterion established by other professionals, has the potential for unintended and even harmful consequences. For example, it may encourage circumvention of formal inter‐professional processes, or more likely, prevent the development of novel collaborations where this labour might require performance.

Although this paper has predominantly focused on challenges at the professional interface, there were accounts of positively orientated interactions that provide a useful direction in terms of addressing foster carers’ and residential carers’ needs. This includes opportunities to meaningfully engage in multi‐agency team meetings and having the distinct expertise of carers being acknowledged and valued. Much of this change will likely be predicated on the reframing and re‐conceptualisation of this social care role as conducting professional work. To this end, continued efforts to support the professionalisation of carers, both as part of formal accreditation and informal status raising may be of help. However, in light of the literature on carers’ professionalisation, it is imperative that this is carefully managed and circumvents the perceptions of control and discipline that has occasionally characterised experiences of this process (Blythe *et al*. 2011, Gilbertson and Barber James 2003, Rosenwald and Bronstein 2008). Furthermore, researchers should consider that effective multi‐agency working is contingent on carers also demonstrating preparedness to acknowledge and accommodate the expertise of other professions, understanding the value of their technical skill when combined with their own experientially acquired knowledge.

Within the context of this study, it is imperative that we consider the extent to which the problematic nature of inter‐professional relationships is bounded by the phenomena of self‐harm. While mainly serving as a departure point for exploration of the wider dynamics of multi‐agency practice, it did provide an initial framing for data collection.

There are a number of particularities to self‐harm management and prevention that encourage reflection to understand the pervasiveness of issues identified presently. Primarily, is the potential for self‐harm, and even suicide, to be constructed as a ‘boundary object’ (Leigh Star 2010, Star and Griesemer 1989). With a clear constructionist orientation, boundary objects encourage us to acknowledge that while some ‘objects’ possess sufficient coherency and meaning across professions so as to facilitate communication, they are characterised by interpretative flexibility. Thus, while professionals are able to find sufficient commonality to permit collaboration, there may be some deep‐rooted incongruence in understandings. The plasticity of the construct of ‘self‐harm’ has been explored previously, with central cleavages orientated to differences between sociocultural and bio‐medical models that are used variously across disciplines and professions (Chandler *et al*. 2011, Evans 2018). As a consequence, it is possible that self‐harm may be particularly susceptible to divergences in beliefs about how expert knowledge is demarcated due to some fundamental differences in understandings about what actually constitutes the phenomena.

Furthermore, it was evident from the data that carers believed themselves to be operating in a context where adolescents’ complex health needs renders them highly visible, which led to marginalisation practices among clinicians. The fact that individuals were presenting with self‐harm only served to amplify this. The sense of adolescents’ stigmatisation permeated how carers understood their own treatment by clinicians, with the notion of courtesy stigma being foregrounded (Goffman 1963). This involves the negative typification of carers, with their close association with the individual in question leading to narratives of blame and responsibility (Corrigan and Miller 2004, Corrigan *et al*. 2006, Pryor *et al*. 2012). It remains questionable whether carers feel similarly stigmatised for other adverse behaviours or outcomes that care‐experienced children and young people might engage in.

As an extension of this issue, is the wider existing literature exploring the predominantly negative discourses and attitudes held by clinicians towards individuals’ engaged in self‐harming practices, perceiving them as making inappropriate or illegitimate claims on medical professionals’ time (Saunders *et al*. 2012, Taylor *et al*. 2009). Within this context, there may be challenges with interacting with clinicians in relation to this area. Yet while such discourses may abound, and may unhelpfully permeate inter‐professional interactions, this characterisation of attitudes needs to be problematised. Arguably, it is too simplistic and reductionist to uncritically draw upon tropes of stigma, blame and illegitimacy. In particular, we need to consider and attend to the complex identity work also being undertaken by clinicians, and whether efforts to exclude care‐experienced individuals and carers’ claims on their expertise may be due to fears about their lack of skill or confidence in this area (Gibb *et al*. 2010, Wilstrand *et al*. 2007).

### Limitations

Data presented in this study are subject to a number of limitations, meaning that the results should be interpreted with caution. First, the purposive sample of a relatively small number of participants limited the generalisability of the data, although in accordance with the grounded theory approach, it generates theoretical propositions that may be taken forward in future research. Second, the study is grounded in carers’ constructions of their identities. Treating the narrator as an interesting sociological construct, we must acknowledge the rhetorical work undertaken in order to build compelling and persuasive accounts of their experiences. Third, data generated in this study are limited by their sole focus on the narratives of carers, and therefore provide an incomplete insight into inter‐professional working within multi‐agency context.

### Implications

The study poses wide‐reaching implications for future research, policy and practice. Most fundamentally we require more nuanced and idiographic research that explore relationships between a more diverse range of professional identities across all realms of social care and healthcare. Researchers need to continue to deconstruct the global identities of ‘social care professional’ and ‘healthcare professional’, attending to the various fractures, hierarchies and tensions at play. There is a considerable literature on inter‐professional practices that can serve as a useful departure point.

There is also a place for further consideration of the specificity of the present findings to the phenomena of self‐harm and suicide, or if these findings reflect a wider set of relational dynamics. As outlined, there are a multitude of reasons why problematic inter‐professional relationships may be a particular consequence of the phenomena in question. Thus, exploration of some of the ideas and concepts progressed in the study with the context of other health areas is warranted. Equally, in order to fully understand how self‐harm and its management may lead to a particular nexus of relationships, notably its existence as a ‘boundary object’ and resultant interpretative flexibility, alternative but complementary study designs are required. Methodologically, research may draw on more ethnographical approaches, while the analytical lenses of discourse and conversation analysis have much to offer in terms of understanding inter‐personal practices *in situ* (Rick 2016).

As a consequence of our current limited insight into inter‐professional working between social care and healthcare within the context of self‐harm, suicide, and mental health more broadly, questions remain about the appropriateness of existing guidance for prevention and management. In particular, recommendations that prescribe multi‐agency approaches are not always supported by a comprehensive and concerted effort to improve the nature and operation of inter‐professional relationships. Resources need to be invested in addressing factors that contribute to poor relationships, such as the issues of contested expertise, professionalisation processes and stigma discussed presently, or the level of job satisfaction, team size and structure discussed elsewhere (O'Carroll *et al*. 2016).

Furthermore, a recent review on inter‐professional working has highlighted how early experiences of IPE at the accreditation stage is associated with positive attitudes to inter‐professional working at a later date (O'Carroll *et al*. 2016). Some efforts have been made to accommodate this evidence, with the Fostering Network recommending the integration of training on the professional role of foster carers into social workers’ educational development (Lawson and Cann 2016). Additional work is required to integrate such approaches across all relevant professional disciplines.

## Declaration of competing interests

The authors declare no potential conflict of interest with respect to the research, authorship and/or publication of this article.

## Funding

The authors disclose receipt of the following financial support for the research, authorship and/or publication of this article: This work was funded by the National Institute for Social Care and Health Research (NISCHR) (SCF‐14‐09) in Wales. The views expressed in this publication are those of the authors and not necessarily those of NISCHR. The work was undertaken with the support of the Centre for the Development and Evaluation of Complex Interventions for Public Health Improvement (DECIPHer), a UK Clinical Research Collaboration Public Health Research Centre of Excellence. Funding from the British Heart Foundation, Cancer Research UK, the Economic and Social Research Council (RES‐590‐28‐0005), the Medical Research Council, the Welsh Government and the Wellcome Trust (WT087640MA), under the auspices of the UK Clinical Research Collaboration, is gratefully acknowledged.

## Data Availability

Research data are not shared due to sensitivity of topic.
